# Endocrine, inflammatory and immune responses and individual differences in acute hypobaric hypoxia in lowlanders

**DOI:** 10.1038/s41598-023-39894-w

**Published:** 2023-08-04

**Authors:** Takayuki Nishimura, Midori Motoi, Hideo Toyoshima, Fumi Kishida, Sora Shin, Takafumi Katsumura, Kazuhiro Nakayama, Hiroki Oota, Shigekazu Higuchi, Shigeki Watanuki, Takafumi Maeda

**Affiliations:** 1 Department of Human Life Design and Science, Faculty of Design, Kyushu University, 4-9-1 Shiobaru, Minami-Ku, Fukuoka, 815-8540 Japan; 2 Department of Living Business, Seika Women’s Junior College, 2-12-1 Minamihachiman, Hakata-Ku, Fukuoka, 812-0886 Japan; 3 Fukuoka Urasoe Clinic, BCC Building 9F, 2-12-19 Ropponmatsu, Cyuou-Ku, Fukuoka, 810-0044 Japan; 4 Department of Medical Laboratory Science, Faculty of Health Sciences, Junshin Gakuen University, 1-1-1 Chikushigaoka, Minami-ku, Fukuoka, 815-8510 Japan; 5 Advanced Testing and Evaluation Center, FITI Testing & Research Institute, 79 Magokjungang 8-ro 3-Gil, Gangseo-gu, Seoul, 07791 South Korea; 6 Department of Anatomy, Kitasato University School of Medicine, 1-15-1 Kitazato, Minami-ku, Sagamihara, Kanagawa 252-0374 Japan; 7 Department of Integrated Biosciences, The University of Tokyo, 5-1-5 Kashiwano-ha, Kashiwa-shi, Chiba, 277-8562 Japan; 8 Department of Biological Sciences, The University of Tokyo, 7-3-1 Hongo, Bunkyo-ku, Tokyo, 113-0033 Japan

**Keywords:** Biochemistry, Biomarkers, Endocrinology, Immunology, Cytokines, Inflammation, Circulation, Kidney, Metabolism, Respiration, Anthropology

## Abstract

When lowlanders are exposed to environments inducing hypobaric hypoxia (HH) such as high mountains, hemodynamic changes occur to maintain oxygen levels in the body. However, changes to other physiological functions under such conditions have yet to be clarified. This study investigated changes in endocrine, inflammatory and immune parameters and individual differences during acute HH exposure using a climatic chamber (75 min of exposure to conditions mimicking 3500 m) in healthy lowlanders. Aldosterone and cortisol were significantly decreased and interleukin (IL)-6, IL-8 and white blood cell (WBC) counts were significantly increased after HH. Lower peripheral oxygen saturation (SpO_2_) was associated with higher IL-6 and WBC counts, and higher IL-8 was associated with higher cortisol. These findings suggest that endocrine, inflammatory and immune responses are evoked even with a short 75-min exposure to HH and individuals with lower SpO_2_ seemed to show more pronounced responses. Our results provide basic data for understanding the physiological responses and interactions of homeostatic systems during acute HH.

## Introduction

In high-altitude environments, the decrease in barometric pressure and consequent fall in partial pressure of oxygen in the alveoli evoke a condition termed “hypobaric hypoxia” (HH). Tibetan and Andean populations are well known to show adaptation to the high-altitude environment and ethnic differences in these adaptations have been identified^1–5^. Typically, Tibetan populations show lower hemoglobin concentrations and high blood flow^2,4,6^, whereas Andean populations show higher hemoglobin concentrations and higher ventilatory features such as barrel chest^2,7^. Each phenotype contributes to maintaining oxygen levels under HH. Conversely, lowlanders exposed to acute or sub-acute HH display several hemodynamic changes (increase in heart rate, ventilation, respiratory rate, cardiac output and hemoglobin concentration) to maintain oxygen levels^8–10^. However, other physiological responses to HH remain poorly understood.

Among lowlanders, acute mountain sickness (AMS) often occurs at altitudes over 3000 m^11,12^, and peripheral oxygen saturation (SpO_2_) has been identified as a predictor of AMS^13–15^. However, other physiological changes occur in the human body. Notably, HH leads to strong natriuresis and diuresis via the reduction of aldosterone^16,17^, and AMS is often associated with fluid retention^17,18^. Many studies have also reported increases in cortisol in response to HH^19–21^, although some studies have found no such changes^21–23^. Adrenaline and noradrenaline responses in HH are more controversial. Noradrenaline appears to increase with chronic HH exposure^23,24^, but with exposure less than around 1 week, no changes are seen in either^24^. In addition, hypoxia and inflammatory responses are known to be related at the molecular, cellular, and clinical levels^25–28^, and inflammatory cytokines such as interleukin (IL)-6, IL-1β and tumor necrosis factor (TNF)-α increase during HH^8,29,30^. In parallel, some studies have reported increases in white blood cell (WBC) count during HH^31,32^. Such findings suggest that not only hemodynamics, but also endocrine, inflammatory and immune responses interact during HH to maintain human homeostasis.

**Figure 1:**
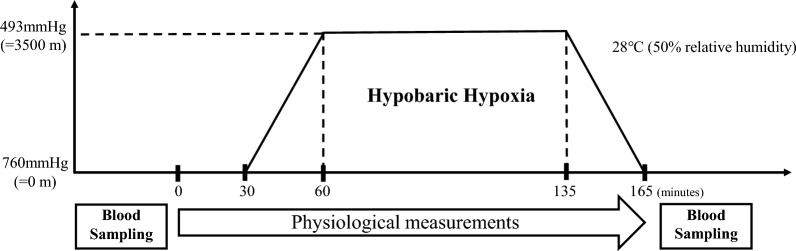
Experimental timeline.

Many studies have reported important data from field research, but factors such as cold, physical burdens, exercise loads and other environmental factors during mountain climbing may well affect physiological responses. In addition, individual differences are seen in physiological responses to HH, including AMS risk^9,11,28^, but such differences have not been fully considered in previous studies. HH is known to evoked various physiological responses, not only in hemodynamic, but also endocrine, inflammatory and immune. Our hypothesis was that individuals showing more pronounced responses in such values would exhibit lower SpO_2_ during HH exposure, reflecting lower blood oxygenation in the body. This study therefore aimed to investigate endocrine, inflammatory and immune responses and individual differences during acute HH in healthy lowlanders using the climatic chamber to reduce confounding factors as much as possible (Fig. 1), to better understand the basic effects of HH on the human body and interactions with human homeostatic systems.

## Results

One-way ANOVA showed that SpO_2_ and HR changed significantly after HH (*p* < 0.001) (Table 1). SpO_2_ was significantly decreased at 60–150 min during HH compared to at 30 min (baseline), while HR was significantly increased at 75–150 min during HH compared to baseline (Table 1). SBP and DBP showed no significant differences between HH and baseline (Table 1).

**Table 1:** Changes over time in physiological data at baseline and with HH exposure.

Altitude (m)	0		0 to 3500		3500					3500 to 0		*p* for ANOVA
Time (min)	15	30	45	60	75	90	105	120	135	150	165	
	Mean (SD)											
SpO_2_ (%)	99.1 (0.8)	98.9 (0.8)	98.3 (1.0)	**94.4 (1.9)**	**87.9 (4.1)**	**86.9 (3.6)**	**86.5 (3.5)**	**85.5 (3.9)**	**86.2 (3.5)**	**90.3 (3.6)**	97.6 (1.4)	< 0.001
HR (beats/min)	67.2 (6.1)	66.6 (6.2)	65.3 (6.5)	70.3 (6.6)	**74.4 (7.8)**	**74.1 (7.5)**	**75.6 (7.6)**	**75.7 (9.0)**	**77.6 (8.8)**	**73.0 (8.2)**	69.1 (7.0)	< 0.001
SBP (mmHg)	–	111.4 (5.6)	110.2 (6.5)	111.3 (5.9)	110.4 (5.0)	110.9 (7.7)	110.1 (6.6)	109.3 (8.1)	109.8 (7.1)	110.9 (8.2)	112 (8.9)	0.553
DBP (mmHg)	–	64 (5.2)	64.8 (6.4)	66.7 (5.2)	66.3 (4.8)	67.3 (6.7)	66.2 (7.2)	66.4 (5.9)	67.1 (6.3)	67.3 (7.0)	65.9 (7.2)	0.115

Bold indicates significant difference (*p* < 0.05) from 30-min data in post hoc test.

**Figure 2:**
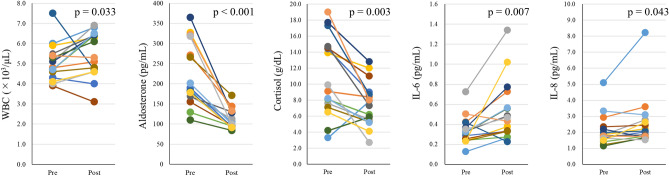
Individual changes in blood components and cytokines. Each color corresponds to an individual subject. After HH, aldosterone and cortisol are significantly decreased (*p* < 0.05) and WBC count, IL-6 and IL-8 are significantly increased (*p* < 0.05).

In terms of blood components, after HH exposure, WBC count was significantly increased (*p* = 0.033) and aldosterone and cortisol were significantly decreased (*p* < 0.001 and *p* = 0.003, respectively) (Table 2, Fig. 2). Noradrenaline was marginally increased after HH exposure (*p* = 0.070) (Table 2). Cytokines IL-6 and IL-8 were significantly increased after HH exposure (*p* = 0.007 and *p* = 0.043, respectively) (Table 3, Fig. 2).

**Table 2:** Changes in blood components and hormone levels.

		Pre		Post		*p*-value
		Mean	SD	Mean	SD	
WBC	(× 10^3^/μL)	**5.1**	**0.9**	**6.0**	**1.5**	**0.033**
RBC	(× 10^4^/μL)	513.5	24.6	516.4	21.3	0.517
Hemoglobin	(g/dL)	15.6	0.8	15.8	0.7	0.358
Hematocrit	(%)	46.5	2.1	46.8	2.0	0.476
MCV	(fL)	90.6	3.3	90.8	3.8	0.383
MCH	(pg)	30.4	1.2	30.5	1.2	0.198
MCHC	(g/dl)	33.6	0.7	33.7	0.7	0.431
Platelet count	(× 10^4^/μL)	26.9	4.4	26.8	4.4	0.778
Adrenaline	(ng/mL)	0.04	0.02	0.04	0.03	0.777
Noradrenaline	(ng/mL)	0.27	0.12	0.35	0.14	0.070
Aldosterone	(pg/mL)	**220.9**	**76.2**	**112.6**	**23.4**	** < 0.001**
Cortisol	(g/dL)	**11.0**	**4.8**	**7.5**	**2.7**	**0.003**

Bold indicates significant difference (*p* < 0.05).

**Table 3:** Changes in cytokine levels.

		Pre		Post		*p*-value
		Mean	SD	Mean	SD	
IFN-γ	(pg/mL)	3.94	4.48	4.12	4.70	0.547
IL-10	(pg/mL)	0.21	0.16	0.22	0.18	0.956
IL-12p70	(pg/mL)	0.12	0.12	0.15	0.14	0.117
IL-13	(pg/mL)	0.77	0.85	0.72	0.93	0.201
IL-1β	(pg/mL)	0.07	0.06	0.08	0.10	0.500
IL-2	(pg/mL)	0.10	0.10	0.11	0.11	0.354
IL-4	(pg/mL)	0.02	0.02	0.02	0.02	0.571
IL-6	(pg/mL)	**0.34**	**0.13**	**0.53**	**0.29**	**0.007**
IL-8	(pg/mL)	**2.10**	**0.95**	**2.56**	**1.57**	**0.043**
TNF-α	(pg/mL)	1.25	0.28	1.25	0.31	0.962

Bold indicates significant difference (*p* < 0.05).

IFN-γ, interferon-γ.

**Figure 3:**
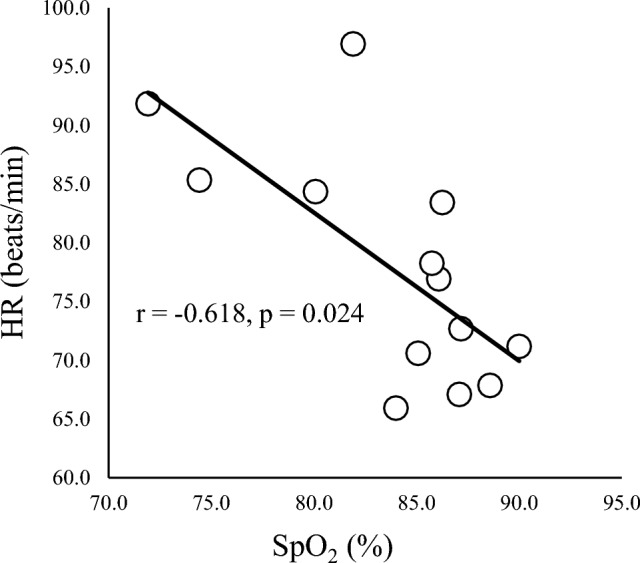
Correlation between SpO_2_ and HR at 135 min. SpO_2_ and HR show a negative correlation (r = − 0.618, *p* = 0.024) at the end of HH (135 min).

SpO_2_ and HR showed significant negative correlations at 75, 105, 120, and 135 min during HH (r = − 0.637, −0.619, − 0.625 and − 0.618, respectively) (Fig. 3).

**Figure 4:**
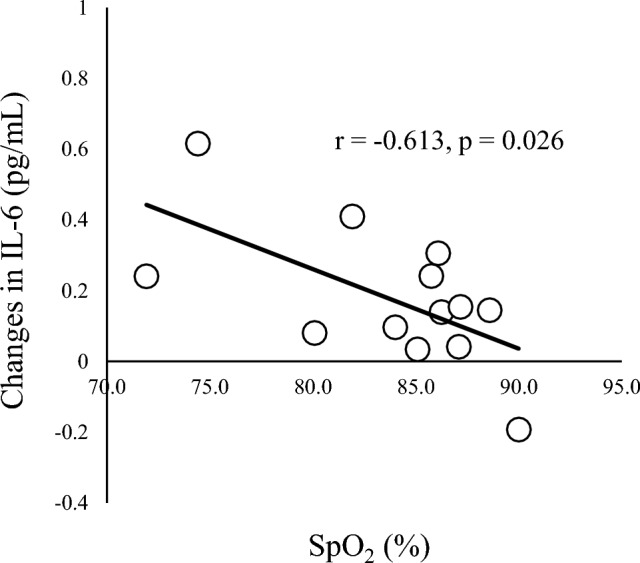
Correlation between SpO_2_ and changes in IL-6. SpO_2_ at 135 min and changes in IL-6 show a negative correlation (r = -0.613, *p* = 0.026).

**Figure 5:**
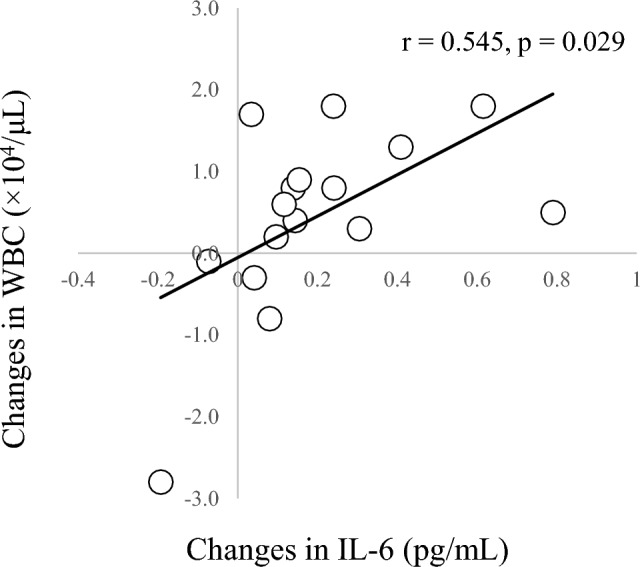
Correlation between changes in IL-6 and WBC count. Changes in IL-6 and WBC counts show a positive correlation (r = 0.545, *p* = 0.026).

**Figure 6:**
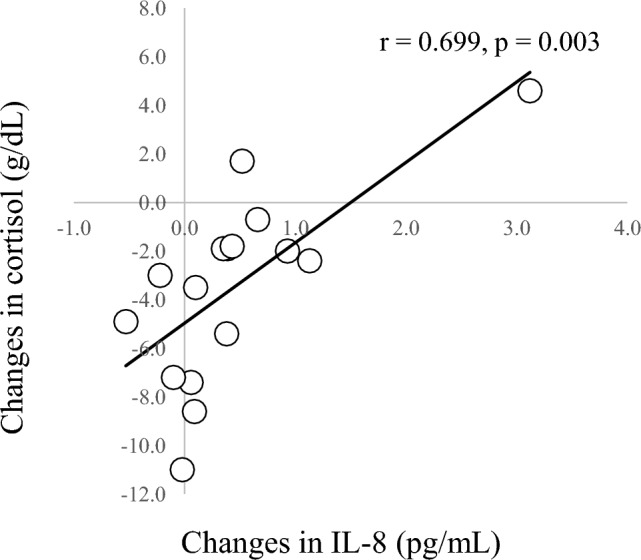
Correlation between changes in IL-8 and cortisol. Changes in IL-8 and cortisol show a positive correlation (r = 0.699, *p* = 0.003).

**Figure 7:**
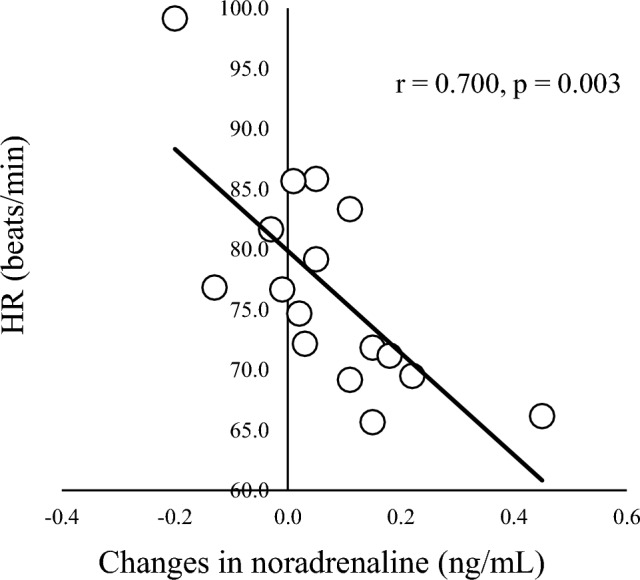
Correlation between changes in noradrenaline and HR at 135 min. Changes in noradrenaline and cortisol show a positive correlation (r = 0.700, *p* = 0.003).

In correlation analyses of other parameters (Table 4), a significant negative correlation was seen between changes in IL-6 and SpO_2_ at 135 min (r = − 0.634, *p* = 0.020; Fig. 4) and a significant positive correlation was seen between changes in IL-6 and changes in WBC count (r = 0.545, *p* = 0.029; Fig. 5). A significant positive correlation was found between changes in IL-8 and changes in cortisol (r = 0.699, *p* = 0.003; Fig. 6). A marginal correlation was noted between changes in WBC count and SpO_2_ at 135 min (r = − 0.513, *p* = 0.073), and a significant negative correlation was identified between changes in noradrenaline and HR at 135 min (r = − 0.700, *p* = 0.003; Fig. 7).

**Table 4:** Correlation coefficients for blood components, cytokines, SpO_2_ and HR.

	Aldosterone	Cortisol	Noradrenaline	IL-6	IL-8	SpO_2_	HR
WBC	0.036	0.406	− 0.149	0.545*	0.100	− 0.513^†^	0.412
Aldosterone	–	0.391	0.077	0.050	0.346	− 0.045	− 0.212
Cortisol	–	–	− 0.043	0.371	0.699**	− 0.247	0.179
Noradrenaline	–	–	–	− 0.061	0.061	0.420	− 0.700**
IL-6	–	–	–	–	0.211	− 0.634*	0.360
IL-8	–	–	–	–	–	0.142	− 0.148
SpO_2_	–	–	–	–	–	–	− 0.618*

Blood data represent amount of change (post–pre) and SpO_2_ and HR data represent values at end of HH (135 min).

**p < 0.01; *p < 0.05; ^†^*p* < 0.1.

## Discussion

SpO_2_ was significantly decreased during HH from 60 to 150 min, while HR was increased from 75 to 150 min. These responses represent typical hemodynamic responses to HH in lowlanders to enhance cardiac output and maintain blood oxygenation^33^. Moreover, SpO_2_ and HR showed negative correlations during HH, indicating higher HR in individuals with lower SpO_2_. These results and associations were consistent with previous studies of lowlanders^9,10,33,34^ and even highlanders^4,5^, and were suggestive of individual differences in some components of adaptability to HH. Although individual variations were seen in hemodynamic responses to HH, subjects were certainly exposed to HH and physiological changes were evoked.

On the other hand, no significant differences in SBP or DBP were seen. Under conditions of hypoxia, peripheral vascular resistance is decreased by nitric oxide released from vascular endothelial cells^35^, which seems to decrease blood pressure, but some studies have reported that blood pressure does not change or is even slightly increased during long and/or severe hypoxic exposure (equivalent to over 4000 m) due to chemoreflex activation of the sympathetic nervous system^36–38^. Responses of blood pressure to HH remain controversial. In this study, blood pressures were unchanged, possibly due to differences in experimental conditions.

All subjects showed decreases in aldosterone after HH, indicating natriuresis and diuresis completely consistent with previous studies^16,17,39^. In addition, cortisol was significantly decreased after HH. In a field study, Wood et al.^21^ reported cortisol at rest was increased over 5150 m, but was unchanged at 3400 m and 4270 m. In addition, cortisol was subdued at 4270 m and increased at 5150 m after exercise. They suggested a threshold of HH effects for cortisol response. Cortisol secretion is stimulated by adrenocorticotropic hormone (ACTH). Mclean^39^ reported decreased levels of salivary cortisol and aldosterone at 4500 m, and assumed that high altitude impaired ACTH stimulation of cortisol and aldosterone secretion. Similarly, Bouissou et al.^40^ reported that the rise in ACTH in response to 60 min of exercise was unchanged in a climatic chamber at 3000 m compared to 0 m, but that cortisol response was subdued. They found that post-exercise ACTH and cortisol levels correlated strongly at 0 m, but not at all during HH, suggesting ACTH-driven steroidogenesis at 0 m, but some disconnect at HH with an apparent reduction in cortisol sensitivity to ACTH. Cortisol is thus increased by stress under severe HH conditions^19,20,22^, whereas mild HH conditions (up to 4000 m) might suppress cortisol secretion due to changes in steroidogenesis or sensitivity to ACTH.

Noradrenaline tended to increase during HH in this study (Table 2). Many previous studies have found no changes in noradrenaline during acute HH^23,24^, but increases with chronic HH (> 1 week)^24,41^. Interestingly, Rostrup et al.^24^ reported that noradrenaline was decreased with 2 days at 4200 m and showed a significant positive correlation between noradrenaline and SpO_2_, suggesting individual variations in acclimatization to HH due to oxygen sensitivity of tyrosine hydroxylase. In the present study, a non-significant tendency toward a positive correlation was seen between noradrenaline and SpO_2_. Furthermore, noradrenaline correlated strongly with HR and SpO_2_ correlated negatively with HR (Table 4, Fig. 7). Although the causal direction of the relationship was unclear, individual variations in HR and SpO_2_ during HH might involve the effects of noradrenaline on α1-adrenoreceptors^42^. Further pharmacological studies are needed to clarify these speculations.

IL-6, IL-8 and WBC counts were increased after HH in this study (Table 3). First, we provided evidence that inflammatory and immune responses were evoked by acute HH even with 75 min of exposure. These results are consistent with previous studies^8,29,30^. IL-6 has various functions^42^, one of which is stimulation of WBC production and immune responses^43^. Similarly, IL-8 is known as a neutrophil chemoattractant^44^ and evokes angiogenesis^45^. IL-6 and IL-8 are thus treated as mediators of inflammation^43,45,46^; as a result, WBC count increased as an immune response in this study. On the other hand, previous studies have reported increases in other cytokines such as IL-1β and TNF-α with longer HH exposures^29,30^. No such changes were seen in this study and this inconsistency might be due to differences in the experimental conditions. In addition, while cytokine levels were significantly increased, the magnitudes of those changes were relatively small, given that values can rise to tens of thousands in infectious diseases. The effects of IL-6 and IL-8 on the biological processes in HH thus need to be considered with care.

Interestingly, individual variations were seen in the responses of IL-6, IL-8 and WBC to HH exposure, with some individuals showing increased values after exposure and others displaying decreased values (Fig. 2). These variations suggest thresholds to HH stimulation because individual variations were seen in relationships between cytokines, cortisol and SpO_2_. The relationship between IL-6 and WBC appears reasonable, with higher IL-6 inducing higher WBC count and immune responses to inflammation. The underlying mechanisms cannot be explained from these data alone, but could involve variations in SpO_2_. Lower SpO_2_ was significantly associated with higher IL-6 and marginally associated with a higher WBC count, suggesting that individuals with lower blood oxygen levels show increased inflammatory and immune responses to HH. In addition, higher IL-8 was significantly associated with higher cortisol (Fig. 6). Cortisol levels mostly decreased after HH (Fig. 2), and this relationship is difficult to explain. However, cortisol has anti-inflammatory effects and increases under IL-6 stimulation^47^, and cortisol exposure is known to increase IL-6 and IL-8 levels in lymphomonocytes^48^. Crosstalk interactions probably exist between cortisol and cytokines, and higher levels of IL-8 might induce secretion of cortisol to mitigate inflammation. IL-6 also showed a similar, non-significant relationship to cortisol. However, the opposite mechanism is another possibility, in that these immune and inflammatory responses may cause lower SpO_2_ via inhibition of hemodynamics. Thus, cellular-level studies are needed to clarify the causal relationships underlying these results.

With these physiological responses, the point of commonality seems to be the lower blood oxygen levels. At the very least, clear associations exist between lower SpO_2_ and higher inflammatory and immune responses. Malacrida et al.^8^ reported that *HIF-1α*, *HIF-2α* and *NRF2* mRNA levels in WBC increased with HH (3830 m) and increased IL-6 levels and oxidative stress. They also reported negative associations between SpO_2_ and IL-6, *HIF-1α*, *HIF-2α* and *NRF2* mRNA levels, and suggested temporal regulation of transcription factors, inflammatory state, and oxidative stress homeostasis in humans during HH. Although we did not measure oxidative stress or levels of HIF-1α or HIF-2α, similar responses seem likely to have occurred in our study. In addition, the factors underlying individual variations in SpO_2_ remain unclear, although some genetic components are likely involved^9,49^. In particular, genetic variations in *HIF-2α* (*EPAS1*) are known to represent a key factor in the low hemoglobin adaptation among Tibetan highlanders^6,50^. HIF-1α and HIF-2α are also known as mediators of inflammatory and immune responses^25,51^. Some genetic variations in *HIF-2α* are seen in the Japanese population and might contribute to SpO_2_ levels and responses to HH.

Taken together, SpO_2_ values are clearly associated with various physiological responses, not only in hemodynamics, but also in hormonal, inflammatory and immune responses even with short HH exposures. While none of our subjects experienced AMS symptoms (LLS score > 3), these responses may precede AMS symptoms. A previous study also reported no association between cytokine levels and AMS^52^. Interestingly, complex physiological interactions and individual variations are apparent in oxygen levels and hormonal, inflammatory and immune responses in the body. Controversial results in previous studies might thus have been due to wide individual variations in such responses to HH. Cytokine storm in coronavirus disease 2019 (COVID-19) has been speculated to involve the secretion of various cytokines, particularly IL-6 as a key mediator^53^. However, in COVID-19, the meta-analysis showed IL‐6 levels of 36.7 pg/mL (95% confidence interval [CI] 21.6–62.3 pg/mL) in severe patients, representing levels markedly lower than those seen in infection diseases such as septic shock (983.6 pg/mL, 95% CI 550.1–1758.4 pg/mL)^54^. Such results suggest that even small increases in IL-6 levels can be physiologically meaningful. The values of cytokines were even lower in the present study, our results suggest that individuals with lower SpO_2_ under HH stress might be at greater risk of cytokine storm, since they may secrete more cytokines in response to hypoxia during lung inflammation. Although we examined some parameters related to SpO_2_ levels, further genetic and transcriptome analyses are needed to clarify the detailed physiological mechanisms and individual differences involved in responses to HH in humans.

This study showed a number of limitations. First, the small sample size limited the ability to analyze individual variations, and more data need to be accumulated. Second, blood was only sampled before and after HH for safety reasons in this study. Some hormone or cytokine levels may thus have rapidly normalized during changes in pressurization. More time points and longer HH exposures are needed for better temporal analyses of physiological responses. Finally, since correlation analyses cannot reveal the direction of causality in a relationship, our ability to interpret the study findings was limited.

In conclusion, cortisol and aldosterone were decreased and HR, IL-6, IL-8 and WBC counts were increased by acute HH. Lower SpO_2_ was associated with higher HR, IL-6 and WBC counts, and higher IL-8 was associated with higher cortisol. These results suggest that inflammatory and immune responses are evoked even with short (75-min) HH and these responses are associated with SpO_2_ levels.

## Methods

### Study subjects

Participants were 16 healthy male students. Mean age was 23.3 (standard deviation [SD] 2.7) years, mean height was 173.3 (6.5) cm, mean weight was 60.1 (5.7) kg and mean BMI was 20.1 (2.2) kg/m^2^. Exclusion criteria were age < 20 years, current smoker status, current use of antioxidant or anti-inflammatory substances at baseline visit, acute illness (infectious, cardiovascular, cerebrovascular or respiratory), or any prior acute high-altitude illness. The Kyushu University Institutional Review Board for Human Genome/Gene Research approved this study protocol and all procedures were carried out in accordance with the approved guidelines. The study is also registered at UMIN (registration no. UMIN000037557). After describing the experimental procedure to potential subjects, written informed consent was obtained prior to enrolment.

### Study protocol

Participants were asked to refrain from eating and drinking 2 h prior to the beginning of the study. Participants wore t-shirts and shorts, and the experiment was conducted while the individual rested in a seated position in a chair. The experimental process is shown in Fig. 1. After blood sampling, various measurement sensors were attached to the participant before the experiment and room temperature was maintained at 28 °C (50% relative humidity). Participants subsequently entered the climatic chamber and physiological measurements were started. After resting for 30 min in an environment at the same air temperature, the programmed operation of the climatic chamber decreased the atmospheric pressure from about 760 mmHg (equivalent to an altitude of 0 m) to 493 mmHg (equivalent to 3500 m). This altitude-equivalent was maintained for 75 min. Atmospheric pressure in the climatic chamber was then increased to 760 mmHg in 30 min and a blood sample was drawn as soon as possible.

### Measurement parameters

Height, weight, and percentage body fat were measured before the experiment. SpO_2_ and heart rate (HR) measurements were sampled at 1-min intervals using a pulse oximeter (Radical-7TM; Masimo Corporation, Tokyo, Japan). Systolic blood pressure (SBP) and diastolic blood pressure (DBP) were measured from the left arm by a digital automatic blood pressure monitor (HEM-7210; OMRON, Kyoto, Japan). SpO_2_ and HR were averaged every 15 min and blood pressure was recorded every 15 min. Lake Louise Score (LLS) was assessed as physiological values were updated to evaluate the risk of AMS^55^. SpO_2_ data were unavailable for 3 subjects because of equipment failure.

Fourteen-milliliter blood samples were drawn from an antecubital vein before and after HH exposure for biochemical analyses. Blood components were measured by standard assay including high-performance liquid chromatography for catecholamines and chemiluminescent immunoassay for steroid hormones (LSI Medience, Tokyo, Japan). Blood cell count (including WBC), hemoglobin, hematocrit, mean corpuscular volume (MCV), mean corpuscular hemoglobin (MCH), MCH concentration (MCHC), platelet count, and levels of adrenaline, noradrenaline, dopamine, aldosterone and cortisol were evaluated. However, most dopamine values were below the limit of detection and were rejected for analysis. Serum cytokine levels were measured using a V-PLEX Proinflammatory Panel 1 human Kit (LSI Medience) to obtain data for interferon-γ, IL-10, IL-12p70, IL-13, IL-1β, IL-2, IL-4, IL-6, IL-8, and TNF-α. Changes in blood components and cytokines were calculated as the value after HH minus the value before HH, and these values were used for correlation analyses.

### Statistical analyses

Physiological data (SpO_2_, HR, SBP and DBP) were compared using one-way analysis of variance (ANOVA) with Tukey post hoc tests. Blood components and serum cytokine levels were compared between pre- and post-HH using Student’s t-test. Pearson product-moment correlation analysis was used to determine relationships between parameters. All data are expressed as mean (SD), with values of *p* < 0.05 considered statistically significant. Because of missing SpO_2_ data, the sample size for data related to SpO_2_ was n = 13. Data were analyzed using Statistical Analysis System software (version 9.4; SAS Institute, Cary, NC, USA).

## Data Availability

The datasets used and analyzed during the current study are available from the corresponding author on reasonable request.

## References

[1] Beall CM (2000). Tibetan and Andean contrasts in adaptation to high-altitude hypoxia. Adv. Exp. Med. Biol..

[2] Beall CM (2006). Andean, Tibetan, and Ethiopian patterns of adaptation to high-altitude hypoxia. Integr. Comp. Biol..

[3] Bigham AW (2013). Andean and Tibetan patterns of adaptation to high altitude. Am. J. Hum. Biol..

[4] Nishimura T, Arima H, Koirala S, Ito H, Yamamoto T (2022). Individual variations and sex differences in hemodynamics and percutaneous arterial oxygen saturation (SpO(_2_)) in Tibetan highlanders of Tsarang in the Mustang district of Nepal. J. Physiol. Anthropol..

[5] Nishimura T (2020). Individual variations and sex differences in hemodynamics with percutaneous arterial oxygen saturation (SpO_2_) in young Andean highlanders in Bolivia. J. Physiol. Anthropol..

[6] Beall CM (2014). Adaptation to high hltitude: Phenotypes and genotypes. Annu. Rev. Anthropol..

[7] Beall CM (1998). Hemoglobin concentration of high-altitude Tibetans and Bolivian Aymara. Am. J. Phys. Anthropol..

[8] Malacrida S (2019). Transcription factors regulation in human peripheral white blood cells during hypobaric hypoxia exposure: An in-vivo experimental study. Sci. Rep..

[9] Motoi M, Nishimura T, Egashira Y, Kishida F, Watanuki S (2016). Relationship between mitochondrial haplogroup and physiological responses to hypobaric hypoxia. J. Physiol. Anthropol..

[10] Naeije R (2010). Physiological adaptation of the cardiovascular system to high altitude. Prog. Cardiovasc. Dis..

[11] Hackett PH, Rennie D, Levine HD (1976). The incidence, importance, and prophylaxis of acute mountain sickness. Lancet.

[12] Schneider M, Bernasch D, Weymann J, Holle R, Bartsch P (2002). Acute mountain sickness: Influence of susceptibility, preexposure, and ascent rate. Med. Sci. Sports Exerc..

[13] Faulhaber M, Wille M, Gatterer H, Heinrich D, Burtscher M (2014). Resting arterial oxygen saturation and breathing frequency as predictors for acute mountain sickness development: A prospective cohort study. Sleep Breath.

[14] Karinen HM, Peltonen JE, Kähönen M, Tikkanen HO (2010). Prediction of acute mountain sickness by monitoring arterial oxygen saturation during ascent. High Alt. Med. Biol..

[15] Roach RC, Greene ER, Schoene RB, Hackett PH (1998). Arterial oxygen saturation for prediction of acute mountain sickness. Aviat. Space Environ. Med..

[16] Bärtsch P (1991). Enhanced exercise-induced rise of aldosterone and vasopressin preceding mountain sickness. J. Appl. Physiol..

[17] Loeppky JA (2005). Early fluid retention and severe acute mountain sickness. J. Appl. Physiol..

[18] Hackett PH (1982). Fluid retention and relative hypoventilation in acute mountain sickness. Respiration.

[19] Martignoni E (1997). The effects of physical exercise at high altitude on adrenocortical function in humans. Funct. Neurol..

[20] Sawhney RC, Malhotra AS, Singh T (1991). Glucoregulatory hormones in man at high altitude. Eur. J. Appl. Physiol. Occup. Physiol..

[21] Woods DR (2012). The cortisol response to hypobaric hypoxia at rest and post-exercise. Horm. Metab. Res..

[22] Benso A (2007). Endocrine and metabolic responses to extreme altitude and physical exercise in climbers. Eur. J. Endocrinol..

[23] Savourey G (1998). Pre-adaptation, adaptation and de-adaptation to high altitude in humans: Hormonal and biochemical changes at sea level. Eur. J. Appl. Physiol. Occup. Physiol..

[24] Rostrup M (1998). Catecholamines, hypoxia and high altitude. Acta Physiol. Scand..

[25] Thompson AA, Binham J, Plant T, Whyte MK, Walmsley SR (2013). Hypoxia, the HIF pathway and neutrophilic inflammatory responses. Biol. Chem..

[26] Singhal R, Shah YM (2020). Oxygen battle in the gut: Hypoxia and hypoxia-inducible factors in metabolic and inflammatory responses in the intestine. J. Biol. Chem..

[27] McGarry T, Biniecka M, Veale DJ, Fearon U (2018). Hypoxia, oxidative stress and inflammation. Free Radic. Biol. Med..

[28] Yasukochi Y, Shin S, Wakabayashi H, Maeda T (2020). Transcriptomic changes in young Japanese males after exposure to acute hypobaric hypoxia. Front. Genet..

[29] Hartmann G (2000). High altitude increases circulating interleukin-6, interleukin-1 receptor antagonist and C-reactive protein. Cytokine.

[30] Iglesias D (2015). Vascular reactivity and biomarkers of endothelial function in healthy subjects exposed to acute hypobaric hypoxia. Clin. Biochem..

[31] Beidleman BA (2006). White blood cell and hormonal responses to 4300 m altitude before and after intermittent altitude exposure. Clin. Sci. (Lond.).

[32] Thake CD, Mian T, Garnham AW, Mian R (2004). Leukocyte counts and neutrophil activity during 4 h of hypocapnic hypoxia equivalent to 4000 m. Aviat. Space Environ. Med..

[33] Ebihara T, Shimizu K, Mitsuyama Y, Ogura H, Oda J (2023). Association between high cardiac output at altitude and acute mountain sickness: Preliminary study on Mt. Fuji. J. Physiol. Anthropol..

[34] Shin S, Yasukochi Y, Wakabayashi H, Maeda T (2020). Effects of acute hypobaric hypoxia on thermoregulatory and circulatory responses during cold air exposure. J. Physiol. Anthropol..

[35] Crawford JH (2006). Hypoxia, red blood cells, and nitrite regulate NO-dependent hypoxic vasodilation. Blood.

[36] Wolfel EE, Selland MA, Mazzeo RS, Reeves JT (1994). Systemic hypertension at 4300 m is related to sympathoadrenal activity. J. Appl. Physiol..

[37] Hansen J, Sander M (2003). Sympathetic neural overactivity in healthy humans after prolonged exposure to hypobaric hypoxia. J. Physiol..

[38] Allemann Y (2004). Impact of acute hypoxic pulmonary hypertension on LV diastolic function in healthy mountaineers at high altitude. Am. J. Physiol. Heart Circ. Physiol..

[39] McLean CJ, Booth CW, Tattersall T, Few JD (1989). The effect of high altitude on saliva aldosterone and glucocorticoid concentrations. Eur. J. Appl. Physiol. Occup. Physiol..

[40] Bouissou P (1989). Effect of beta-adrenoceptor blockade on renin-aldosterone and alpha-ANF during exercise at altitude. J. Appl. Physiol..

[41] Mazzeo RS, Wolfel EE, Butterfield GE, Reeves JT (1994). Sympathetic response during 21 days at high altitude (4300 m) as determined by urinary and arterial catecholamines. Metabolism.

[42] Kamimura D, Ishihara K, Hirano T (2003). IL-6 signal transduction and its physiological roles: The signal orchestration model. Rev. Physiol. Biochem. Pharmacol..

[43] Kaplanski G, Marin V, Montero-Julian F, Mantovani A, Farnarier C (2003). IL-6: A regulator of the transition from neutrophil to monocyte recruitment during inflammation. Trends Immunol..

[44] Hammond ME (1995). IL-8 induces neutrophil chemotaxis predominantly via type I IL-8 receptors. J. Immunol..

[45] Li A, Dubey S, Varney ML, Dave BJ, Singh RK (2003). IL-8 directly enhanced endothelial cell survival, proliferation, and matrix metalloproteinases production and regulated angiogenesis. J. Immunol..

[46] Nielsen AR, Pedersen BK (2007). The biological roles of exercise-induced cytokines: IL-6, IL-8, and IL-15. Appl. Physiol. Nutr. Metab..

[47] Steensberg A, Fischer CP, Keller C, Møller K, Pedersen BK (2003). IL-6 enhances plasma IL-1ra, IL-10, and cortisol in humans. Am. J. Physiol. Endocrinol. Metab..

[48] Da Pozzo E, Giacomelli C, Cavallini C, Martini C (2018). Cytokine secretion responsiveness of lymphomonocytes following cortisol cell exposure: Sex differences. PLoS ONE.

[49] Yasukochi Y, Nishimura T, Motoi M, Watanuki S (2018). Association of EGLN1 genetic polymorphisms with SpO_2_ responses to acute hypobaric hypoxia in a Japanese cohort. J. Physiol. Anthropol..

[50] Beall CM (2010). Natural selection on EPAS1 (HIF2alpha) associated with low hemoglobin concentration in Tibetan highlanders. Proc. Natl. Acad. Sci. U. S. A..

[51] Taylor CT, Colgan SP (2017). Regulation of immunity and inflammation by hypoxia in immunological niches. Nat. Rev. Immunol..

[52] Swenson ER (1997). Acute mountain sickness is not altered by a high carbohydrate diet nor associated with elevated circulating cytokines. Aviat. Space Environ. Med..

[53] Ghanem M, Brown SJ, Eat Mohamed A, Fuller HR (2022). A meta-summary and bioinformatic analysis identified interleukin 6 as a master regulator of COVID-19 severity biomarkers. Cytokine.

[54] Leisman DE (2020). Cytokine elevation in severe and critical COVID-19: A rapid systematic review, meta-analysis, and comparison with other inflammatory syndromes. Lancet Respir. Med..

[55] Sutton, J. R. In *International Hypoxia Symposium* (Pergamon Press, 1991).

