# Author Correction: Endocrine, inflammatory and immune responses and individual differences in acute hypobaric hypoxia in lowlanders

**DOI:** 10.1038/s41598-023-40794-2

**Published:** 2023-08-22

**Authors:** Takayuki Nishimura, Midori Motoi, Hideo Toyoshima, Fumi Kishida, Sora Shin, Takafumi Katsumura, Kazuhiro Nakayama, Hiroki Oota, Shigekazu Higuchi, Shigeki Watanuki, Takafumi Maeda

**Affiliations:** 1 Department of Human Life Design and Science, Faculty of Design, Kyushu University, 4‑9‑1 Shiobaru, Minami‑Ku, Fukuoka, 815‑8540 Japan; 2 Department of Living Business, Seika Women’s Junior College, 2‑12‑1 Minamihachiman, Hakata‑Ku, Fukuoka, 812‑0886 Japan; 3 Fukuoka Urasoe Clinic, BCC Building 9F, 2‑12‑19 Ropponmatsu, Cyuou‑Ku, Fukuoka, 810‑0044 Japan; 4 Department of Medical Laboratory Science, Faculty of Health Sciences, Junshin Gakuen University, 1‑1‑1 Chikushigaoka, Minami‑ku, Fukuoka, 815‑8510 Japan; 5 Advanced Testing and Evaluation Center, FITI Testing & Research Institute, 79 Magokjungang 8‑ro 3‑Gil, Gangseo‑gu, Seoul, 07791 South Korea; 6 Department of Anatomy, Kitasato University School of Medicine, 1‑15‑1 Kitazato, Minami‑ku, Sagamihara, Kanagawa 252‑0374 Japan; 7 Department of Integrated Biosciences, The University of Tokyo, 5‑1‑5 Kashiwano‑ha, Kashiwa‑shi, Chiba, 277‑8562 Japan; 8 Department of Biological Sciences, The University of Tokyo, 7‑3‑1 Hongo, Bunkyo‑ku, Tokyo, 113‑0033 Japan

Correction to: *Scientific Reports* 10.1038/s41598-023-39894-w, published online 04 August 2023

The original version of this Article contained an error in Figure 2 where the data for Aldosterone (pg/mL) was incorrect. The original Figure 2 and accompanying legend appear below.

**Figure 2:**
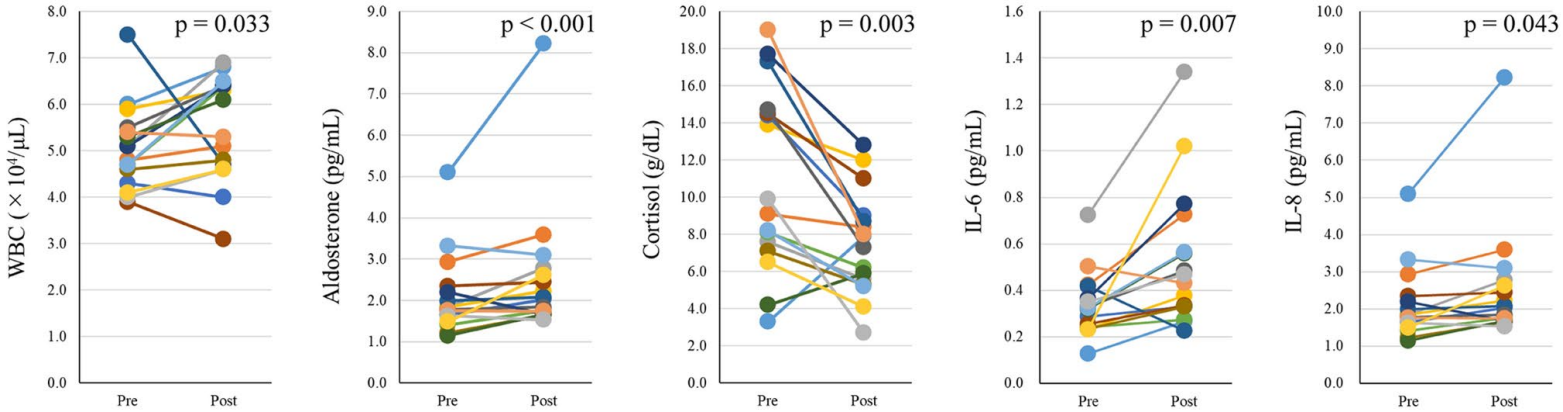
Individual changes in blood components and cytokines. Each color corresponds to an individual subject. After HH, aldosterone and cortisol are significantly decreased (*p* < 0.05) and WBC count, IL-6 and IL-8 are significantly increased (*p* < 0.05).

The original Article has been corrected.

